# Supplementary material for: Genome Wide Identification of Sesame Dof Transcription Factors and Functional Analysis of SiDof8, SiDof10 and SiDof34 in Fatty Acid Synthesis
Source: Curr Issues Mol Biol. 2025 Aug 30;47(9):700. doi: 10.3390/cimb47090700 (PMC12468942; doi:10.3390/cimb47090700)
Supplement: Supplementary file 1 [file cimb-47-00700-s001.zip › cimb-3710706-supplementary.pdf]

Table S1. qRT-PCR primer information of sesame Dof genes.

Gene	Forward primer sequence	Reverse primer sequence
SiDof1	GCCCACCAACAATTAGGCCA	AGGCTGAGAGAGGCTGTAGT
SiDof2	TTGATGGGCTTGGGAGCAAT	TCAGTGCTTGTGTTGCTGGA
SiDof3	TTCTGCATCCCACTGTCGTC	AGGCGAGTCAGGACCAAAAG
SiDof4	CCTCTCCCAGCCCAGATACT	GACGACGAGGGGATGTTGTT
SiDof5	ATCAGCTCCACCGTCGTCTA	ACTGCCCCACGAAATTCTCT
SiDof6	GGAGCAACAAGCGAACCAAG	CTCTTCGTCTCCTCCGCTTC
SiDof7	TTGGATCAGGTCGTCGCAAA	CAAAGTTGCGTGTCAGAGCC
SiDof8	TGCAGGAGGCACAGATCAAAA	AGGAACCCCCTTCAGTCCAA
SiDof9	CGAAGCGCAGAGAGAACGTA	CGAAGTTAGGGCTTGCGTTG
SiDof10	GTGACTCCACCGACACCAAA	GATACTGATGCCGATGCGGA
SiDof11	AGTAATGGGTGTGGAGTGCG	AAGGTCCGGCCATGAAAAGT
SiDof12	AGGCCAGTTGAAAACCCAAGA	TGGGACGTTCCTCAAAGTGC
SiDof13	TGCGACTCCACAAACACCAA	CGCTCATCTTACCCTCGCTC
SiDof14	TCCTCTGCCGTTCTACTCCA	GCCGGCTTCAAGATGTTGTC
SiDof15	CCAGGTGCAAAAGCATGGAAA	AAATAAGCAGCCCTCCGACA
SiDof16	TCAATGCCCACAGAAGCAGA	AAAAGTAGCGTGGCTGGGAT
SiDof17	ATCACCCGCACTTCCATCTC	CCAAAGAAGGCAAACACGCA
SiDof18	TTGAACTGTCCGAGGTGTGG	GTGGGGTGTGAGATCTTGGG
SiDof19	AAAACAGGGAGAACGGCGAT	GTTTACAGTGGGAGACGGCA
SiDof20	TTCTTCAAGCGCACAACACC	GCTGGGAGAGGCTGTAGTTG
SiDof21	AAGCCCTCAAATGCCCAAGA	CTCCTCCAACCGGGACATTC
SiDof22	CGCTGTGGTTCTTCCAACAC	ATTCCTGAGGGACCCACCTT
SiDof23	GAAACAGCCCTCAAATGCCC	CAAGCCCATATCGCTGGTGA
SiDof24	AACAAATCTCAGCCTCGCCA	TCATGCCGTCTGTCGTTTCA
SiDof25	GGCATCAATCAAGGGGTTGC	TTGTGGCTCCTGTGGTTTCA
SiDof26	ACAACTACAGCCTCACGCAG	TGGAGTCCTTTGAGTCGCAG
SiDof27	TACCCTGCTTTTCTGGAGCG	GTACGGCACAACCCCAGTAA
SiDof28	CGGACCCGAGTGAAAGGAAG	GCGGCATGATTTGCAGAAGT
SiDof29	TGATTCGCCCAACACCAAGT	TGGTGATTGAAGCCCACCAA
SiDof30	GTCGCCGCAAAACTAAGCAG	GGCCATAAAAAGGTGCTCCG
SiDof31	CGAAACATCCCTGTCGGAGG	GTGTCGTGGGCACTGTTTTT
SiDof32	AAAGTCTCCGGCCAGTAACG	ATTCCTCCTGCACCACCAAG

SiDof33	CTCATCACCAGCCCCTCAAG	GGTTCCCCCTCTAGTCCAGT
SiDof34	CTCTCTACCCCGGTTTGTGG	GGACGTTTCTCAAGGCTCCA
SiActin	CTGAGAGATTCCGCTGTCCG	AAGGTGCTGAGGGATGCAAG

Table S2. Vector constructing primer information of *SiDof8*, *SiDof10* and *SiDof34*.

Name	Forward primer sequence	Reverse primer sequence
SiDof8-OE	GGATCCATGGATACTGCTCAGTGGCC	GTCGACTCACCATGAACCTCCAGCC
SiDof10-OE	GGATCCATGCAGAATTCATCATTTTACTCAC	GTCGACCTACTGAAAATTTGAGGAAGGTG
SiDof34-OE	GGATCCATGATTCAAGAGCTGTTGGG	GTCGACTTAAGGGTAAGCACCATTGG
SiDof8-GFP	GGATCCATGGATACTGCTCAGTGGCC	GTCGACCCATGAACCTCCAGCC
SiDof10-GFP	GGATCCATGCAGAATTCATCATTTTACTCAC	GTCGACCTGAAAATTTGAGGAAGGTG
SiDof34-GFP	GGATCCATGATTCAAGAGCTGTTGGG	GTCGACAGGGTAAGCACCATTGG

Table S3. Physicochemical properties and subcellular localization of sesame Dof proteins

Gene name	Gene ID	Scaffolds	Amino number (aa)	Molecular weight (Da)	Isoelectric point	Subcellular localization
SiDof1	LOC105155434	LG2:4460597-4461491	297	32901.17	4.47	nucleus
SiDof2	LOC105156034	LG2:13201155-13203258	302	33057.69	8.16	nucleus
SiDof3	LOC105157099	LG3:2687825-2691185	496	53022.55	5.55	nucleus
SiDof4	LOC105157219	LG3:4086777-4088887	331	36358.31	6.45	nucleus
SiDof5	LOC105157727	LG3:8645692-8647584	238	24921.17	8.20	nucleus
SiDof6	LOC105158041	LG3:11375397-11377042	299	32125.22	5.98	nucleus
SiDof7	LOC105159975	LG4:3971559-3974952	430	47595.85	6.06	nucleus
SiDof8	LOC105160319	LG4:8930645-8932697	306	33367.83	8.68	nucleus
SiDof9	LOC105162358	LG1:10741879-10743237	302	31986.02	6.17	nucleus
SiDof10	LOC105163550	LG1:11671178-11672335	236	24637.58	5.78	nucleus
SiDof11	LOC105166214	LG7:4440877-4442130	251	26842.10	8.63	nucleus
SiDof12	LOC105166761	LG7:9184408-9186268	252	28381.57	9.28	nucleus
SiDof13	LOC105167467	LG8:2620143-2620506	215	22148.64	8.77	nucleus
SiDof14	LOC105167652	LG8:3493723-3497108	497	53362.02	5.39	nucleus
SiDof15	LOC105167857	LG8:7821690-7822487	159	17966.22	9.12	nucleus
SiDof16	LOC105167892	LG8:8013199-8014375	327	35413.22	6.98	nucleus
SiDof17	LOC105167893	LG8:8016741-8018498	278	30339.57	9.71	nucleus
SiDof18	LOC105168584	LG8:8188138-8192695	305	33262.06	6.49	nucleus
SiDof19	LOC105168787	LG8:14948732-14962111	497	53562.26	5.57	nucleus
SiDof20	LOC105170336	LG9:2530015-2532115	319	34313.90	7.06	nucleus
SiDof21	LOC105170685	LG9:5204173-5206567	291	31809.44	8.09	nucleus
SiDof22	LOC105174207	LG11:11136758-11137603	281	30317.95	6.52	nucleus
SiDof23	LOC105174708	LG11:13978256-13980059	329	34738.72	9.56	nucleus
SiDof24	LOC105175506	LG12:4028693-4030704	293	32239.93	7.61	nucleus
SiDof25	LOC105175553	LG12:4336714-4338983	295	31950.77	9.72	nucleus
SiDof26	LOC105176376	LG13:3815962-3817981	341	37095.26	8.96	nucleus
SiDof27	LOC105177260	LG15:1006200-1019695	510	55383.27	5.60	nucleus
SiDof28	LOC105177758	LG15:3345196-3346399	233	24408.25	8.51	nucleus
SiDof29	LOC105178230	LG15:8227375-8228953	338	36430.69	9.00	nucleus
SiDof30	LOC105179502	unplaced scaffold	155	17285.58	9.65	nucleus
SiDof31	LOC105179111	unplaced scaffold	335	36494.26	8.75	nucleus
SiDof32	LOC105161465	LG5:793197-795227	333	35055.48	9.50	nucleus

SiDof33	LOC105175554	LG12:4345162-4347121	350	38226.03	8.14	nucleus
SiDof34	LOC105177686	LG15:3268756-3269854	335	35130.09	8.91	nucleus

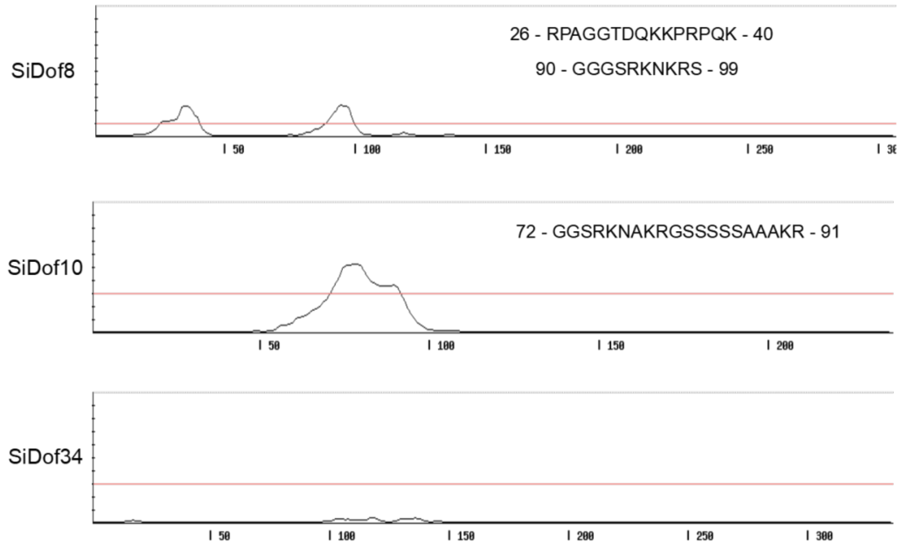

Figure S1. Nuclear localization signal prediction of SiDof8, SiDof10 and SiDof34 in NLStradamus (http://www.moseslab.csb.utoronto.ca/NLStradamus/).
